# Pharmacists’ Activities to Reduce Medication Waste: An International Survey

**DOI:** 10.3390/pharmacy6030094

**Published:** 2018-08-29

**Authors:** Charlotte L. Bekker, Helga Gardarsdottir, Antoine C. G. Egberts, Marcel L. Bouvy, Bart J. F. van den Bemt

**Affiliations:** 1 Department of Pharmacy, Sint Maartenskliniek, 6574 NA Nijmegen, The Netherlands; c.bekker@maartenskliniek.nl; 2 Department of Clinical Pharmacy, Division Laboratories and Pharmacy, University Medical Centre Utrecht, 3584 CX Utrecht, The Netherlands; h.gardarsdottir@uu.nl (H.G.); a.c.g.egberts@umcutrecht.nl (A.C.G.E.); 3 Division of Pharmacoepidemiology and Clinical Pharmacology, Utrecht Institute for Pharmaceutical Sciences, Utrecht University, 3584 CG Utrecht, The Netherlands; m.l.bouvy@uu.nl; 4 Department of Pharmacy, Radboud University Medical Centre, 6525 GA Nijmegen, The Netherlands; 5 Department of Clinical Pharmacy and Toxicology, Maastricht University Medical Centre, 6229 HX Maastricht, The Netherlands

**Keywords:** medication waste, unused medication, pharmacy practice, clinical pharmacy, survey research

## Abstract

**Aim:** To identify activities that pharmacists undertake to reduce medication waste, and to assess the extent to which these activities are implemented, their importance for waste-reduction and feasibility for broad implementation. **Methods:** A two-phase survey was conducted among community and hospital pharmacists working in different developed countries. Phase one used an open-ended questionnaire to identify activities undertaken by pharmacists. Answers were thematically analysed to construct a list of medication waste-reducing activities. In phase two, a questionnaire was disseminated among pharmacists from different countries, to assess if these activities are implemented (yes/no), their importance and feasibility (1 to 5 ranking scale). **Results:** In phase one, 53 pharmacists participated and 14 activities were identified. These were categorized into the pharmaceutical supply chain: prescribing, dispensing (pharmacy/patient-related) and leftover stage. In phase two, 89 pharmacists participated. Most activities were implemented by a minority of pharmacists. Reducing medication amounts in stock was most frequently implemented (dispensing stage pharmacy-related; 86%), followed by collecting unused medications (leftover stage; 77%) and performing a medication review (dispensing stage; 68%). Waste-reducing activities in the dispensing stage activities were both considered most important and feasible (ranked 4). Overall, most activities scored higher on importance than on feasibility. **Conclusions:** Pharmacists have various opportunities to reduce medication waste throughout the pharmaceutical supply chain, however, not all are broadly implemented. Pharmacists consider waste-reducing activities important, but they are less certain about the feasibility for implementation in practice.

## 1. Introduction

Medication waste can occur in all stages of the pharmaceutical supply chain. For instance, physicians may prescribe unnecessarily large quantities (prescribing stage). During the dispensing stage, pharmacists dispense larger quantities as manufacturers’ package sizes may exceed the amount required for treatment. Once medication has been supplied to the patient, early treatment changes, for example, due to some side effects or unsatisfactorily efficacy, can lead to an excessive amount of unused medication at home. Moreover, low adherence of patients to treatment regimens can contribute to medication waste as well. Finally, medications that are left unused and of good quality, are generally destroyed if returned to the pharmacy [1,2,3,4,5].

There is increased awareness of the financial impact of medication waste [6,7,8,9]. Health care budgets are limited and unused medications can be considered a waste of resources. It is important that patients dispose of these properly, for instance, by returning these to pharmacies or chemical waste depots. However, patients sometimes incorrectly dispose of unused medications through household garbage, the toilet, or sink, with the risk of polluting the environment [10]. Active pharmaceutical ingredients have been detected in surface, ground, and drinking water [11,12] that may have detrimental effects on aquatic species and ecosystems [13,14]. Efforts to reduce medication waste and the undesirable economic and environmental burden are, therefore, warranted.

Pharmacists are key players in the pharmaceutical supply chain and are in a position to contribute to the reduction of medication waste [15]. One can presume that individual pharmacists have already initiated various strategies to reduce this waste. However, information about activities that are implemented in practice to reduce waste is limited. The availability of such information could facilitate an exchange of knowledge between pharmacists on how to reduce medication waste and could promote the implementation of such activities in daily practice. Therefore, the aim of this study was to identify activities that individual pharmacists have currently undertaken in community and hospital pharmacies in developed countries to reduce medication waste. Moreover, this study aimed to assess the extent to which these activities are implemented, the importance of the activities for reducing waste, and the feasibility for broadly implementing these activities in daily practice. 

## 2. Materials and Methods 

### 2.1. Study Design 

**Figure 1:**
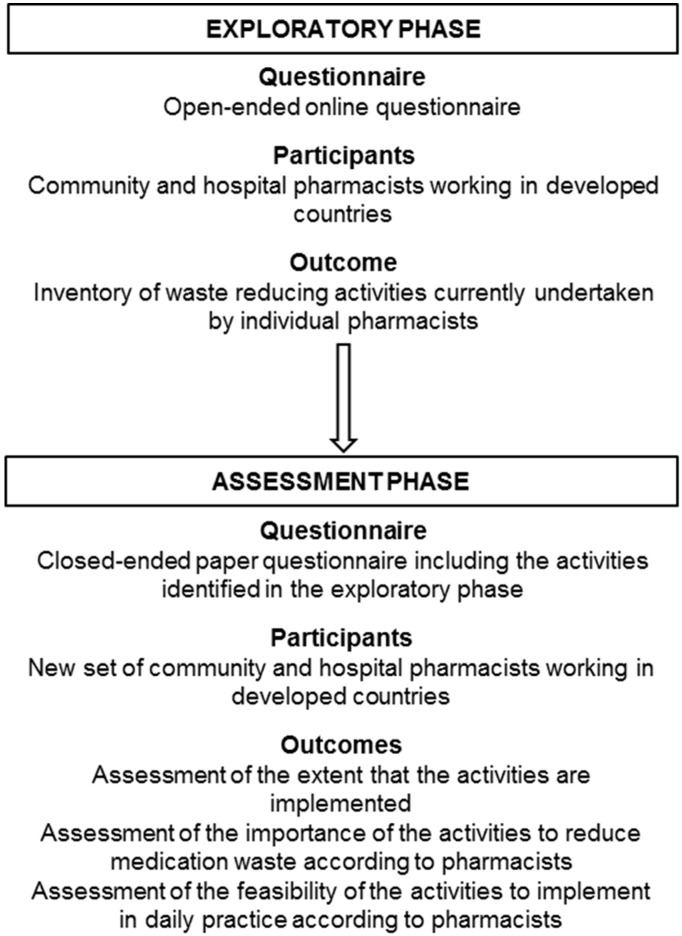
Overview of the main methods used for the two phases.

This survey consisted of two phases: an exploratory phase of which the results were used for the subsequent assessment phase. The study was conducted between July 2014 and October 2016. The first phase aimed to identify activities currently undertaken by individual pharmacists and the second phase aimed to assess the extent to which these activities are implemented and their importance and feasibility (see Figure 1 for overview).

### 2.2. Ethics

All data were analysed anonymously. Under Dutch law, no approval from an Ethical Review Board was required as only health care professionals were involved.

### 2.3. Phase One: Exploration

#### 2.3.1. Participants’ Inclusion and Data Collection

Pharmacists working in a community, hospital or academic setting located in a country with a ranking of ‘very high‘ on the human development index [16] were eligible for participation. Pharmacists were approached through (inter)national organizations of pharmacists or through the personal network of the research group. Pharmacists received an email invitation explaining the purpose of the study that included a link to the questionnaire. Non-responders received two reminders, the first reminder was sent two weeks after the initial invitation and the second two weeks thereafter. Countries were only included in the analysis if two pharmacists from that country completely filled in the questionnaire.

#### 2.3.2. Questionnaire

Activities that individual pharmacists have implemented to reduce medication waste were explored by an open-ended questionnaire that was created in an online survey tool. The questionnaire was developed by the research group and pre-tested in terms of interpretation by a pharmacist who was not involved in the study. The questionnaire consisted of three sections (see Appendix A): the prescribing, the dispensing and the leftover stage. Each section consisted of several questions that focused on activities implemented to reduce medication waste. As the community and hospital setting may differ, hospital pharmacists were asked two additional questions regarding activities implemented during the (preparation prior to) administration of medications and activities implemented at the hospitals’ wards. Pharmacists’ country of origin and work setting (community pharmacy/hospital pharmacy/academic) were recorded as well. 

#### 2.3.3. Data Analysis 

Data from the questionnaires were exported to Microsoft Excel version 2010 (Microsoft, Albuquerque, NM, USA) and analysed using thematic content analysis [17]. Pharmacists’ answers were coded by the first researcher and reviewed by the second researcher [18]. Any disagreements between the two researchers were discussed until consensus was reached. Hereafter, both researchers independently categorized the activities into the three previous defined stages according to their content which they subsequently discussed until both agreed.

### 2.4. Phase Two: Assessment of Implementation, Importance and Feasibility 

#### 2.4.1. Participants’ Inclusion and Data Collection

A questionnaire was constructed based on the results of the first phase. This questionnaire was distributed among pharmacists participating in the 45th European Symposium on Clinical Pharmacy that was held in Oslo, Norway, in October 2016. Only questionnaires completed by pharmacists working in a country, as defined in phase one, were included in the analysis.

#### 2.4.2. Questionnaire

Questions were formulated for all activities that were identified during the first phase of the study and divided into the predefined stages (Appendix B). The questionnaire was also pre-tested by a pharmacist not involved in the research study. For each activity, pharmacists were asked to indicate whether the activity was implemented in their country (yes/no), to rank the importance of the activity to reduce waste and the feasibility to implement in practice. Answers were measured on a scale with a range from one, denoting the activity as not important or feasible, to five, very important or feasible. In addition, pharmacists were able to add other activities if these were not included. Their country of origin and work setting (community pharmacy/hospital pharmacy/academic/other) were recorded as well. 

#### 2.4.3. Data Analysis

Data from the questionnaires were imported in Microsoft Excel and descriptively analysed (frequencies and percentages). To equally weigh the frequency scores, more than 50% of the pharmacists within a country should have reported implementing the activity, because activities taken by fewer than half of the pharmacists within a country were assumed to be taken at random and therefore not counted. The importance and feasibility ranking scales were assessed as medians with interquartile ranges. First, the median ranking for each activity within each country was determined. Subsequently, the median ranking for all activities were calculated and averaged per stage. All analyses were performed in STATA version 13 (StataCorp, College Station, TX, USA) and Microsoft Excel.

## 3. Results

Fifty-three pharmacists from 19 developed countries were included in the first phase of the study (Appendix C). The activities currently undertaken by individual pharmacists to reduce medication waste were categorized into the prescribing, dispensing and leftover stage. During the analysis, two subthemes within the dispensing stage were added, i.e., activities related to the pharmacy or to the patient’s medication therapy and storage practices. In total, 14 main activities were identified (Table 1).

Eighty-nine pharmacists from 22 developed countries were included in the second phase (Appendix D). The pharmacists reported no new activities on top of the activities that were identified in phase one. Results of the two phases are presented together per stage hereafter to facilitate a comprehensive presentation.

### 3.1. The Prescribing Stage

To reduce medication waste in the prescribing stage, two main activities that were undertaken were identified. Namely, prescribers could tailor the prescribed amount and pharmacists could counsel prescribers on the prescribed amount. Most pharmacists mentioned that prescribers tailor the amount based on medication characteristics (e.g., cost), on patient characteristics (e.g., age) and the expected duration of time until symptoms should resolve. Some pharmacists remarked that they counsel prescribers on how to prevent waste. For instance, by recommending the duration of use for each prescription whenever possible.

**Figure 2:**
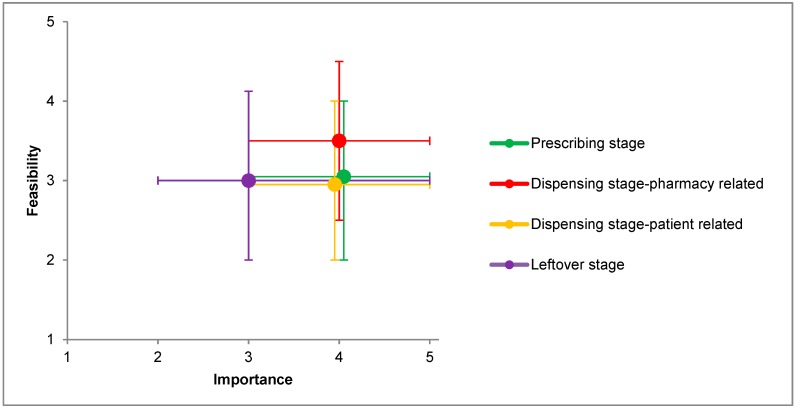
For each stage of the pharmaceutical supply chain, median importance ranking to reduce medication waste and median feasibility ranking to implement in practice (with upper and lower quartile) given by the pharmacists.

**Figure 3:**
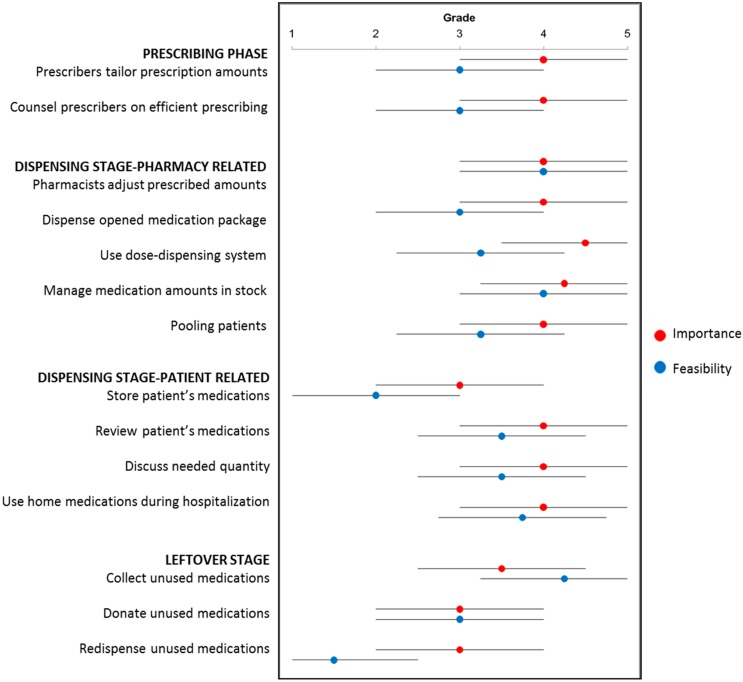
For each activity, median importance ranking to reduce medication waste and median feasibility ranking to implement in practice (with upper and lower quartile) given by the pharmacists.

Activities in the prescribing stage were reported to be implemented by approximately one-third of the countries (Table 1). On average, these activities were considered important for reducing waste (median ranking 4), and were ranked neutral in terms of the feasibility of their implementation in practice (median ranking 3, Figure 2 and Figure 3).

### 3.2. The Dispensing Stage

#### 3.2.1. Pharmacy Related Activities

Activities undertaken by pharmacists to reduce medication waste in the dispensing phase focused mainly on dispensing smaller amounts to the patient, by adjusting the amount of prescribed medications to the treatment duration, dispensing opened medication packages and using dose-dispensing systems. Most pharmacists indicated that the number of days for which medications can be dispensed is limited by law and generally concerns a three-month supply. Some pharmacists mentioned that they are allowed to adjust the amount of medications prescribed without consulting the prescriber. One example of such an activity is when a pharmacist notices that a physician has prescribed more than needed, they inform the patient and reduce the dispensed amount. However, this approach is not achievable for all pharmacists as it was frequently reported that pharmacists are only allowed to dispense complete medication packages, even when the prescribed amount is less. Concerning internal waste management at the pharmacy, pharmacists mentioned that they manage the amount of medications kept in stock. For example, some pharmacies exchange medications that are rarely used or that are close to the expiry date to prevent disposal. In some hospital pharmacies, patients who are treated with parenteral medications are scheduled on the same day in order to pool injection vials. 

Stock management was most frequently reported activity implemented to reduce medication waste, in 86.4% of the responding countries. Of these countries, 94.7% indicated that they limit the amount of medications that are kept in stock and 73.7% collaborated with other pharmacies to exchange medications. The other pharmacy-related activities of the dispensing stage were reported to be implemented by approximately half of the countries. The activities were ranked the highest in terms of importance and feasibility. Of all activities, using dose-dispensing systems and stock management ranked highest concerning their importance for reducing waste (median ranking > 4), but lower on feasibility for implementation (median ranking 3 and 4 respectively).

#### 3.2.2. Patient-Related Activities

Patient-related activities for reducing waste reported in the dispensing stage aimed at optimizing medication therapy and storage management by the patient. These include storing the majority of patient’s medications at the pharmacy, reviewing the patient’s medications, and starting a dialogue with the patient about the quantity needed. Furthermore, through discussion with the patient, pharmacists try to adjust the dispensed amount to the patient’s actual needs, and to increase their awareness about waste. Some hospital pharmacists reported that patients are allowed to use their own home medications during hospital admission, thereby reducing medication waste. 

Sixty-eight percent of the responding countries reported to perform medication reviews. Only 9.1% of the countries stored patients’ medications at the pharmacy and this was considered less feasible (median ranking 2). Overall, patient-related activities in the dispensing stage were considered important for reducing waste (median ranking 4), but scored lower on feasibility for implementation (median ranking 3).

### 3.3. The Leftover Stage

Three waste-reducing activities were identified in the leftover stage. Community and hospital pharmacists mentioned that the amount of unused medications is collected in the pharmacy for safe disposal. A few pharmacists indicated that these medications are donated to charities for people in need. As a last activity, hospital pharmacists mentioned that unused medications were redispensed, under the condition that the medications were stored at the hospital ward and had not been dispensed to patients. 

Of the responding countries, 77.3% reported collecting unused medications and 18.2% donating unused medications. None of the countries reported redispensing unused medications returned by patients. Activities aimed at tackling medication waste during the leftover stage scored lowest in terms of both importance and feasibility (median rankings 3).

## 4. Discussion

This study shows that pharmacists undertake several activities to limit medication waste in all stages of the pharmaceutical supply chain. More than half of the participating countries reported using dose-dispensing systems, managing the amount of medication in stock, performing medication reviews, and collecting unused medications. Pharmacists considered activities of the prescribing and dispensing stage most important for reducing medication waste and pharmacy-related activities of the dispensing stage most feasible for implementation in practice. Most activities scored lower in terms of feasibility than importance. 

This is the first study that gives an overview of activities taken by community and hospital pharmacists. For this study, several limitations could be identified. Most importantly, only pharmacists were consulted. It is possible that other healthcare professionals would identify other medication waste-reducing activities. Also, not all pharmacists of the countries approached responded to the survey, hence, some activities might have been missed. However, no additional activities were mentioned in the second phase of the study that included other countries as well. Therefore, one can assume that the list of potential activities to reduce waste is comprehensive. Third, the second researcher was not blinded for the coding of the first researcher. However, the pharmacists mentioned concrete activities and thus the risk of misclassification is considered minimal. Fourth, not all questionnaires were fully completed. We found that the reported answers of uncompleted questionnaires did not differ from the fully completed questionnaires. Hence, it is assumed that the missing answers would not have altered the findings. Fifth, the respondents and the activities they reported might not necessarily be representative for their whole country. However, it still enabled us to report on activities that pharmacists have implemented to reduce medication waste and to indicate which activities are implemented most frequently. Sixth, only activities implemented by the majority of pharmacists within a country were considered to be implemented by that country. This could have resulted in an underestimation of the frequency that activities were taken. Finally, this study involved pharmacists working in developed countries, and any generalization of our results with respect to other countries should be viewed with caution. 

Many pharmacists considered the waste-reducing activities as important, which emphasizes the necessity for interventions that aim to combat medication waste. The study suggests that activities that are related to the organization of the pharmacy and the dispensing stage were most often implemented and were considered most feasible. Overall, activities that focus on waste prevention were found to be most promising. But as not all activities were considered achievable to implement in practice, this may suggest that barriers hamper feasible implementation and a need for feasible waste-reducing interventions. Looking at the current evidence of potential interventions, an example of a waste-reducing activity in the prescribing stage is to dispense smaller amounts of expensive medications. Limiting the amount of medication supplied for a first time to a two-week period, followed by 30 days for a repeat prescription [19], may decrease the risk of unused medications and unnecessary waste. Patients receiving medications for more than 30 days are more likely to waste a part of those medications [20,21]. Additionally, pharmacists could also supply a trial prescription amount to the patient at the start of treatment and supply the remainder when the medication is well tolerated. Paterson et al. showed that a split-fill supply could reduce the cost of medication waste [22]. Regarding the dispensing stage, studies show that increasing the frequency of medication batch preparations or scheduling patients with the same therapy on the same day in the hospital pharmacy could reduce medication waste and expenditures [23,24,25]. However, applying such strategies in the community pharmacy is not financially feasible as large quantities of relatively low-cost medications are generally dispensed and additional dispensing fees may outweigh the savings on medication costs [26]. Pharmacists should, therefore, consider the individual medication costs when deciding if smaller amounts should be dispensed to the patient, as this may not always save costs, however, it might still reduce the risk of environmental pollution.

It is important not to focus on waste reduction by prescribers and pharmacists but also to increase patients’ awareness of medication waste. Patients often only pay a part of the medication cost out of pocket and are not always aware of the total cost of medication. Governments and health care authorities have started campaigns to raise patients’ awareness about medication prices, including displaying the price on the medication package or on the dosage label [27]. Furthermore, discussing the quantity dispensed with the patient could reduce the supply of unwanted medications and, potentially, medication waste [28]. If adherence of patients to their treatment regimen could be increased, medication waste might be reduced as well. Moreover, medication reviews could be periodically conducted to identify medication therapies that are dispensed to patients but no longer needed or non-adherence. Unnecessary medication therapies could thereby be discontinued helping to reduce the waste of unnecessary healthcare costs. Regarding the leftover stage, very few interventions have been investigated and most studies assess the amount and economic value of medications returned to pharmacies [2,4,29,30,31]. The donation of medications to other countries is disapproved of by the World Health Organization [32]. The question as to whether medications returned to pharmacies could be redispensed remains hypothetical [33,34], as many prerequisites need to be addressed in order to redispense unused medications, such as how to ascertain the quality of the medications, the patients trust in redispensed medications, and the legal- and financial feasibility [35,36].

Multiple interventions seem promising for reducing medication waste. However, it seems that various barriers hamper their implementation. Barriers one could think of are each nation’s reimbursement systems which influence how medications are prescribed, dispensed and collected at the pharmacy. Furthermore, legislation could be challenging to the implementation of waste-reducing activities. Some of the respondents reported that different activities, such as splitting packages into smaller quantities, are not legally allowed. Even within a country, pharmacists can counteract waste differently as this will also depend on the availability of resources in the pharmacy, like sufficient knowledge of pharmacy workers of the possibilities to reduce medication waste and the monetary budget. For the successful implementation of waste-reducing interventions, such barriers should be identified and overcome first.

## 5. Conclusions

This study demonstrates that pharmacists have developed many activities to reduce medication waste in all stages of the pharmaceutical supply chain. However, not all potential activities to reduce medication waste have been implemented in daily practice. Activities focusing on waste prevention seem most promising. Even though pharmacists consider activities for reducing medication waste important, they are less certain about the feasibility of broadly implementing these activities in daily clinical practice.

## Figures and Tables

**Table 1:** Waste-reducing activities that can be undertaken by pharmacists and their estimated frequency of activities implemented to reduce medication waste. A country was considered to have implemented an activity if more than 50% of the pharmacists within that country reported that the activity is implemented.

Activity	Countries (n = 22) n (%)
**The prescribing stage**	
Prescribers tailor prescription amounts	7 (31.8)
Counsel prescribers on efficient prescribing	7 (31.8)
**The dispensing stage**	
*Pharmacy related*	
Pharmacists adjust prescribed amounts	10 (45.5)
Dispense opened medication package	11 (50.0)
Use dose-dispensing system	12 (54.5)
Manage medication amounts in stock	19 (86.4)
Limiting storage amounts	18 (94.7)
Exchange medications with other pharmacies	14 (73.7)
Pooling patients	7 (31.8)
*Patient related*	
Store patient’s medications	2 (9.1)
Review patient’s medications	15 (68.2)
Discuss needed quantity	5 (22.7)
Use home medications during hospitalization	10 (45.5)
**The leftover stage**	
Collect unused medications	17 (77.3)
Donate unused medications	4 (18.2)
Redispense unused medications	0 (0)

## Appendix A. Questionnaire of Phase One

1. Which initiatives are taken nationally and in pharmacies to minimize medication waste?
  ▪ Initiatives taken by prescribers.
  ▪ Initiatives taken in the pharmacy during dispensing.
  ▪ Initiatives taken for leftover unused medications.

Prescribing stage

2. Which phases are taken by prescribers to minimize medication waste?
3. In which cases is the number of drugs prescribed tailored on a patient’s health condition?
4. In which cases is the number of drugs prescribed tailored on costs or on other drug characteristics?

Dispensing stage

5. Which initiatives are taken in pharmacies during the dispensing of medications?
6. What is the maximum amount of days for which medications can be dispensed in your country?
7. Which initiatives are taken at the different departments of the hospital?
8. Which initiatives are taken in the hospital pharmacy while preparing/compounding the medication?

Leftover stage

9. What is done with unused medications that are returned to the pharmacy?

## Appendix B. Questionnaire of Phase Two

	**Activity Taken in Your Country**		**Important for Decreasing Medication Waste**	**Feasible to Implement in Practice**
	**Yes**	**No**	**1 (Not)–5 (Very)**	**1 (Not)–5 (Very)**
**The prescribing stage**				
Do prescribers tailor the amount of medications that they prescribe in order to limit medication waste?				
Do pharmacists counsel physicians on how to combat potential medication waste?				
**The dispensing stage**				
Are pharmacists allowed to adjust the prescribed amount during dispensing in order to limit potential medication waste?				
Are pharmacist allowed to remove medication from the original package in order to limit potential medication waste (e.g., split packages)?				
Are pharmacists using (unit) dose dispensing systems?				
Are pharmacists managing the amount of medications that is kept in stock in the pharmacy in order to limit medication waste? If yes, how? ○ Limiting the amount that is kept in stock. ○ Collaborating with other pharmacies to exchange almost expired medications. ○ Other:				
Are pharmacists in the hospital pharmacy scheduling patients on the same day so that medication is prepared at once in order to limit medication waste (e.g., pooling of patients with IV drugs)?				
Please explain other actions taken during dispensing that limit medication waste:				
**Optimizing stock management by the patient**				
Are pharmacists enabling patients to store a part of their prescribed medications in the pharmacy?				
Are pharmacists reviewing patient’s medications in order to limit medication waste (e.g., medication review, optimization of pharmacotherapy)?				
Are pharmacists discussing the quantity needed for symptom improvement with the patient in order to limit medication waste (increase awareness about waste)?				
Are patients allowed to bring their home medication to the hospital and use this during hospitalization?				
Please explain other actions discussed during patient’s counselling that limit medication waste:				
**The leftover stage**				
Are pharmacists collecting unused medications so they can dispose of them safely?				
Are pharmacists allowed to donate unused medications that are returned to the pharmacy to other countries or people in need?				
Can unused medications that are returned to pharmacies by patients be re-dispensed to a different patient? ○ If yes, do the returned medications need to apply with specific criteria? Which criteria?				

## Appendix C. Number of Pharmacists per Country of Origin and Work Setting that Participated in Phase One

**n = 19**	**Total n = 53**	**Community Pharmacy n = 39**	**Hospital Pharmacy n = 14**
Australia	2	2	
Belgium	4	3	1
Canada	2	2	
Croatia	2	2	
Denmark	3	2	1
Estonia	5	4	1
Finland	2	1	1
France	2	1	1
Iceland	8	8	
Ireland	2	1	1
Italy	2		2
Malta	2	1	1
Netherlands	4	3	1
New Zealand	2	1	1
Norway	2	1	1
Spain	2	2	
Switzerland	3	2	1
United Kingdom	2	2	
United States	2	1	1

## Appendix D. Number of Pharmacists per Country of Origin and Work Setting That Participated in Phase Two. 12 Pharmacists Were Working in Multiple Settings and therefore the Total Sum Exceeds 89

**n = 22**	**Total n = 89**	**Community Pharmacy n = 13**	**Hospital Pharmacy n = 66**	**Academic n = 15**	**Other n = 7**
Australia	1		1		
Austria	11		11	1	
Belgium	2		2		
Canada	2	2		1	
Croatia	1	1			
Denmark	1	1		1	
Estonia	2		2		
Finland	1				1
France	6		6	2	
Germany	2	2		1	
Ireland	1			1	
Italy	2		2		
Netherlands	5	2	1	1	1
Norway	24		21	2	1
Portugal	4	1	2	1	1
Romania	2		2		
Slovakia	1		1		
Slovenia	4		3	1	
